# Immune Suppression and Rapid Invasion of Nile Tilapia Gills Following an Acute Challenge by *Flavobacterium davisii*

**DOI:** 10.3390/biology13110894

**Published:** 2024-11-02

**Authors:** Yingxuan Xu, Shifeng Wang, Yongcan Zhou, Zhenyu Xie, Bei Wang, Zhangding Zhao, Wenlong Cai, Peibo Wang, Weiliang Guo, Dongdong Zhang, Zhi Ye

**Affiliations:** 1 Hainan Provincial Key Laboratory for Tropical Hydrobiology and Biotechnology, School of Marine Biology and Fisheries, Collaborative Innovation Center of Marine Science and Technology, School of Breeding and Multiplication (Sanya Institute of Breeding and Multiplication), Hainan University, Haikou 570228/Sanya 572025, China; violet135180@163.com (Y.X.); cwhs3@hainanu.edu.cn (S.W.); zychnu@163.com (Y.Z.); xiezyscuta@163.com (Z.X.); peibowang@hainanu.edu.cn (P.W.); guowl07@mails.jlu.edu.cn (W.G.); 2 Provincial Key Laboratory of Aquatic Animal Disease Control and Healthy Culture, College of Fishery, Guangdong Ocean University, Zhanjiang 524088, China; wangb@gdou.edu.cn; 3 Hainan Baolu Aquatic Products Technology Co., Ltd., Haikou 570208, China; zzd@progift.net.cn; 4 Department of Infectious Diseases and Public Health, Jockey Club College of Veterinary Medicine and Life Sciences, City University of Hong Kong, Kowloon Tong, Hong Kong SAR, China; wenlocai@cityu.edu.hk; 5 MOE Key Laboratory of Marine Genetics and Breeding, College of Marine Life Sciences Ocean University of China, Qingdao 266071, China; 6 Key Laboratory of Tropical Aquatic Germplasm of Hainan Province, Sanya Oceanographic Institution, Ocean University of China, Sanya 572025, China

**Keywords:** *Flavobacterium davisii*, Nile tilapia, gill, immune response, RNA-seq

## Abstract

*Flavobacterium davisii* is one of the causative agents of columnaris disease, significantly impacting Nile tilapia aquaculture. This study examines the invasion and immune evasion mechanisms of a highly virulent *F. davisii* strain through transcriptomic profiling of tilapia gills following acute immersion. We identified 8192 differentially expressed genes (DEGs) at 2 h, 6 h, and 12 h post-infection. They are enriched in pathways related to oxidative stress, immune suppression, tissue necrosis, and bacterial infection. Notably, early overexpression of rhamnose-binding lectin and mucin genes facilitated bacterial adhesion. Key immune genes, including those encoding major histocompatibility complex (MHC), immunoglobulins (Ig), Toll-like receptors (TLRs), and chemokines, were downregulated, indicating immune suppression. Conversely, immune evasion genes such as Fc receptor-like (FcRL) and programmed death-ligand 1 (PDL1) were upregulated, along with genes associated with reactive oxygen species (ROS) production, leading to increased tissue damage. Additionally, the upregulation of fibroblast growth factor and collagen genes suggested active tissue repair. In conclusion, *F. davisii* rapidly invades its host by enhancing adhesion to gill tissues, suppressing immune function, and inducing tissue damage. These findings enhance our understanding of *F. davisii* infection mechanisms and support the future breeding of disease-resistant tilapia and the development of sustainable control strategies.

## Simple Summary

Nile tilapia are particularly susceptible to columnaris disease caused by *Flavobacterium davisii,* with the mucosal gill and skin tissues serving as the primary infection routes. This study investigates the mechanisms by which this highly virulent strain invades tilapia, focusing on immune responses in gill tissues post-infection. We found that, during the early stages of infection, genes that promote bacterial adhesion were significantly upregulated, while crucial immune-related genes were downregulated, compromising the fish’s immune defenses. This immune suppression allows the bacteria to cause extensive tissue damage. By elucidating the molecular mechanisms underlying adhesion and immune evasion, our findings enhance the understanding of columnaris disease pathogenesis in tilapia. This knowledge will aid in developing disease-resistant breeding programs and contribute to more effective and environmentally sustainable disease management strategies in aquaculture.

## 1. Introduction

Aquaculture continues to grow faster than other food production sectors, with Nile tilapia (*Oreochromis niloticus*) being one of the most important freshwater aquaculture species in worldwide tropical and subtropical regions. However, tilapia aquaculture faces several challenges, especially disease-causing pathogens such as bacteria. Infection in fish is a dynamic interaction between the pathogen and the host. After entering the fish’s mucus layer, bacteria begin to aggregate and proliferate while simultaneously using adhesins, flagella, and pili to contact and bind to epithelial cells in the mucosal tissue through cell surface receptors [1]. When bacteria adhere to epithelial cells, the related signaling pathways within the host cells are activated, triggering a series of defense responses. These responses include elevating the cholesterol content in cell membranes to make them more robust, killing pathogens through phagocytosis, and initiating other innate and adaptive immune reactions [2,3]. Bacteria, on the other hand, employ various mechanisms to evade host immune surveillance and clearance [4,5,6] and can even use the host’s immune responses to enhance their adhesion [7,8,9,10]. The outcome of this host-pathogen interaction determines the pathogen’s ability to invade the host internally and cause substantial damage [2]. Therefore, pathogen adhesion to the host is the first step towards successful infection and is critical for effective intervention. 

The first point of bacterial adhesion to the fish is the mucosal barrier, which is in continuous contact with the external environment, exposing fish to many waterborne pathogens. Among these pathogens is *Flavobacterium columnare*, the causative agent of columnaris, which causes a serious threat to freshwater cultured fishes [11], including tilapia [12,13,14,15]. Research has shown that the adhesion of *F. columnare* to gill and skin tissues, particularly gill mucosal tissue, is crucial to its pathogenicity. The early hours of infection are critical for adhesion and invasion, with highly virulent strains more likely to adhere to gill tissues than less virulent strains [16,17,18,19]. Following colonization in the gills and skin, *F. columnare* induces characteristic clinical signs, including skin lesions, gill damage, fin rot, and high mortality, especially in juvenile fish [16].

According to the latest taxonomic and nomenclature rules, *F. columnare* has been categorized into four new species: previous genotype I stains retained the name *F. columnare*, while genotypes II, III, and IV have been renamed *F. covae* sp. nov., *F. davisii* sp. nov., and *F. oreochromis* sp. nov., respectively [19,20]. In our preliminary work, a highly virulent *Flavobacterium* strain (HNU-01) isolated from Nile tilapia and belonging to genotype III was designated as *F. davisii* [14]. The subsequent immersion infection of Nile tilapia with *F. davisii* revealed that the gills had the highest bacterial load compared to other tissues. However, our understanding of the specific molecular mechanisms underlying *F. davisii* adhesion to the host remains limited, and there is a lack of in-depth functional studies targeting the genes involved in these mechanisms. Therefore, this study investigated the changes in immune response in gill tissues following an acute immersion challenge with *F. davisii* to explore bacterial invasion and infection mechanisms. These findings not only provide knowledge to help researchers understand the pathogenesis of columnaris disease in Nile tilapia, but also contribute to future efforts in breeding disease-resistant fish and developing effective and environmentally friendly control strategies. 

## 2. Materials and Methods

### 2.1. Fish Husbandry, Infection, and Sample Collection

In this experiment, 300 healthy Nile tilapia with an average weight of approximately 60 ± 1.0 g were temporarily housed in aquaria in a flow-through system for two weeks. They were randomly divided into control and infection groups, with three 200 L replicate tanks each (50 fish per replicate). All fish were sourced from the Baolu Aquatic Products Technology Co., Hainan Province, China. Upon transportation to the laboratory’s rearing facility, fish were acclimated to fresh water at 29–30 °C for two weeks. Feed (Tongwei, China) was withheld for 24 h before the experiment started, during which fish behavior was observed, and water quality parameters were regularly monitored to ensure they remained within normal ranges. These parameters included 6–7 mg/L dissolved oxygen, pH 7.6, and non-detectable ammonia and nitrites. 

All procedures of fish handling and treatment during this study were approved by the Hainan University Institutional Animal Care and Use Committee (HNU-IACUC) before initiation. Before the challenge, gill specimens were collected from six randomly selected fish for PCR detection to confirm they were *F. davisii*-free. The challenge experiments were then conducted by immersing the fish for 1 h at a final concentration of 2.5 × 10^8^ CFU/mL of *F. davisii* strain HNU-01 [14]. Briefly, the preserved culture of the F. davisii HNU-01 isolate was sub-cultured twice on modified Shieh agar. A single colony was then inoculated into MS broth and cultured at 28 °C with shaking at 125 rpm (to avoid clumping) for 12–24 h. Subsequently, a portion of the culture was transferred to fresh MS broth for further propagation and adjusted to a final concentration for infection. The control fish were immersed in the MS broth following the same protocols as the treatment group but without bacteria. After the challenge, fish were incubated in aquaria in a flow-through system, and the water was exchanged every two days. Gills were collected at 0 h (control), 2 h, 6 h, and 12 h following pathogen infection. Five fish were randomly selected from each replicate tank of control and infected groups at each timepoint, and gill samples were then collected and pooled together, generating one sample per tank (three samples per group) for each timepoint. All samples were stored at −80 °C until processing. The five experimental fish displayed the characteristic clinical signs of columnaris disease, and the bacteria were confirmed by re-isolation from diseased fish as described in previous studies [14,20]. Briefly, their gill samples were flushed with sterile PBS and homogenized in sterile PBS by vigorous agitation with a grinding bar for 1 min. The homogenates were then plated on MS agar and incubated at 28 °C for 24 to 36 h. Dominant uniform colonies were randomly selected for PCR and subsequent sequencing to confirm their identity.

### 2.2. RNA Extraction, Library Preparation, and Sequencing

Total RNA was extracted from the gill tissue using TRIzol® Reagent (Invitrogen, Carlsbad, CA, USA) according to the manufacturer’s instructions. Then, RNA quality was determined by a 5300 Fragment Analyzer (Agilent Technologies, Inc., Santa Clara, CA, USA) and quantified using the ND-2000 (Thermo Fisher Scientific, Waltham, MA, USA). Only high-quality RNA samples (OD260/280 = 1.8~2.2, OD260/230 ≥ 2.0, RIN ≥ 6.5, 28S:18S ≥ 1.0, > 1μg) were used to construct the sequencing library. Reverse transcription was performed using HiScript IV RT SuperMix Kit from Vazyme (Nanjing, China). Library construction and sequencing were performed at Shanghai Majorbio Bio-pharm Biotechnology Co., Ltd. (Shanghai, China) according to the kit manufacturer’s instructions (Illumina, San Diego, CA). The tilapia gill RNA-seq transcriptome library was prepared following Illumina® Stranded mRNA Prep, Ligation from Illumina (San Diego, CA, USA) using 1 μg of total RNA. Firstly, mRNA was isolated using the polyA selection method by oligo (dT) beads and then fragmented by fragmentation buffer. Secondly, double-stranded cDNA was synthesized using a SuperScript Double-Stranded cDNA Synthesis Kit (Invitrogen, Carlsbad, CA, USA) with random hexamer primers (Illumina, San Diego, CA, USA). Then, cDNA was subjected to end-repair, phosphorylation, and ‘A’ base addition following Illumina’s library construction protocol. Libraries were size selected for cDNA target fragments of 300 bp on 2% Low Range Ultra Agarose (Bio-Rad Laboratories, Hercules, Ipswich, CA, USA) followed by PCR amplification using Phusion DNA polymerase (NEB, MA, USA) for 15 PCR cycles. After quantification by Qubit 4.0 Fluorometer (Thermo Fisher Scientific, Waltham, MA, USA), the paired-end RNA-seq library was sequenced with the NovaSeq 6000 sequencer (2 × 150 bp read length).

### 2.3. Quality Control and Reads Mapping

The raw paired-end reads were trimmed and quality controlled by fastp [21] with default parameters. Then, clean reads were separately aligned to the reference genome (GCF_001858045) with orientation mode using HISAT2 software [22]. The mapped reads of each sample were assembled by StringTie [23] in a reference-based approach. 

### 2.4. Differential Expression and Enrichment Analysis

The expression level of each transcript was calculated according to the transcripts per million reads (TPM) method to identify differentially expressed genes (DEGs) between the two different treatments. RNA-Seq by Expectation Maximization (RSEM) [24] was used to quantify gene abundances. Differential expression analysis was performed using the DESeq2 [25]. DEGs with fold change ≥ 2 and False Discovery Rate (FDR, adjusted *p*-value) ≤ 0.05 (DESeq2) were considered significant. In addition, functional enrichment and pathway analysis, including Gene Ontology (GO) and Kyoto Encyclopedia of Genes and Genomes (KEGG), were performed to identify which DEGs were significantly enriched in GO terms and metabolic pathways at Bonferroni-corrected *p*-value ≤ 0.05 compared with the whole-transcriptome background. GO functional enrichment and KEGG pathway analysis were carried out by GOATOOLS and KOBAS [26], respectively.

### 2.5. qPCR Validation

According to functional enrichment and pathway analysis results of RNA-seq, twelve of the significant expressed genes with different patterns were randomly selected to be validated by qPCR. Their corresponding primer sequences are provided in the Supplementary section (Table S1). For this validation, we utilized cDNA that was 10-fold diluted from the same tissue sample used for the initial transcriptome sequencing. Real-time PCR was performed using 2 × ChamQ Universal SYBR qPCR Master Mix (Vazyme, Nanjing, China) for fluorescence-based quantification. The qPCR cycling conditions were as follows: an initial denaturation at 95 °C for 30 s, followed by 40 cycles of denaturation at 95 °C for 5 s, annealing at 60 °C for 30 s, and extension at 72°C for 10 s. A final extension step was carried out at 72 °C for 10 s. After qPCR completion, a melt curve analysis was conducted to ensure the specificity of the amplified products. Each experimental group underwent triplicate testing. Expression data from the qPCR analysis were processed using the 2^−ΔΔCt^ method for relative quantification [27]. All data are presented as the mean ± standard error (SE). *p*-values ≤ 0.05 were considered statistically.

## 3. Results

### 3.1. F. davisii Infection

**Figure 1:**
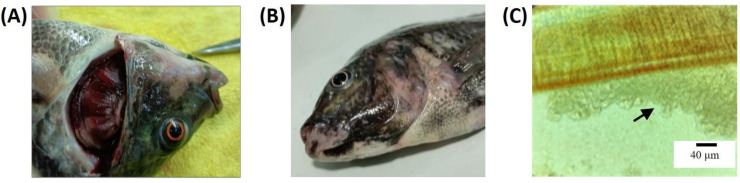
Gill and skin lesions induced by *Flavobacterium davisii* in Nile tilapia. (**A**) Hemorrhaging and ulceration in the gill filaments. (**B**) Skin discoloration. (**C**) Yellowish, grass-like bacterial colonies on the gill filament as indicated by the arrow.

Fish in the infected group exhibited lesions characteristic of columnaris (Figure 1), including hemorrhaging and ulceration in the gill filaments and scaling discoloration on the skin. Additionally, the microscopic examination of the gill filaments revealed yellowish, grass-like bacterial colonies. Pathogen isolation was performed by culturing tissues from the lesions on modified Shieh agar, and PCR confirmation identified the causative agent as *F. davisii* [20].

### 3.2. Sequencing Summary

The initial experiments using the NovaSeq 6000 sequencer produced a total of 83.63 Gb of 150 bp clean reads after filtering the raw data. The clean data for each sample exceeded 7.37 Gb, with the Q30 base percentage surpassing 93.62%, a GC content of approximately 50%, and an average error rate well below 0.05% (Table S2). After filtering and aligning the reads to the Nile tilapia genome, the total expression differences among samples were minimal, indicating the high reliability of the sample data. Raw read data were archived at the NCBI Sequence Read Archive (SRA) under the Accession Number SRP440770.

### 3.3. Identification of Differentially Expressed Genes (DEGs) and Enrichment Analysis

**Figure 2:**
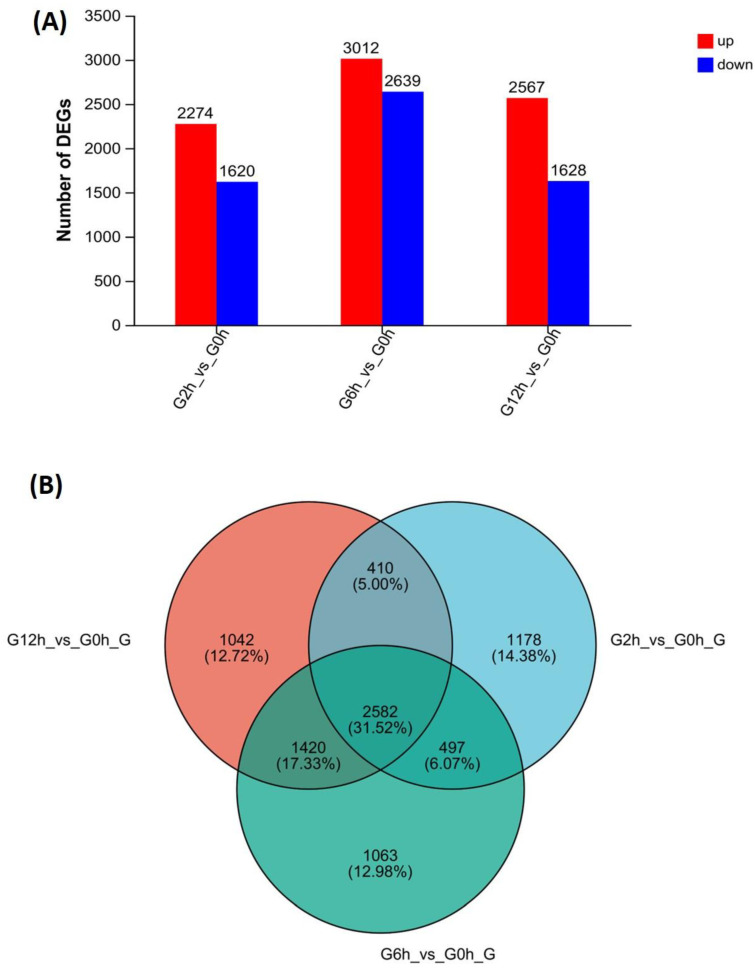
Differentially expressed genes (DEGs) in Nile tilapia gills acutely challenged with *Flavobacterium davisii*. (A) The number of upregulated and downregulated DEGs in the gills at 2, 6, and 12 h post-challenge compared with the control (0 h). (B) The number of shared DEGs in the gills at 2, 6, and 12 h post-challenge compared with the control (0 h).

Differential expression analysis identified 8192 DEGs in the infected group compared to the healthy controls, using the criteria of a fold change ≥ 2 and FDR ≤ 0.05 (Figure 2; Table S3). The number of DEGs at each timepoint post-challenge was 3894 (2 h), 5651 (6 h), and 4195 (12 h), respectively, with 2582 genes shared among the infected groups.

**Figure 3:**
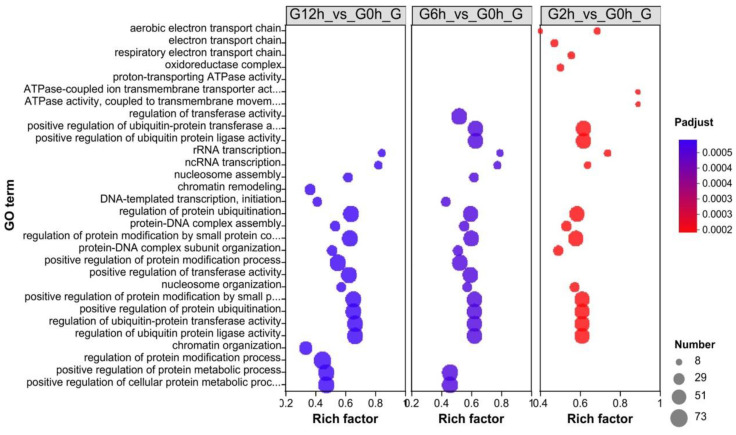
Significantly enriched Gene Ontology (GO) terms identified through enrichment analysis of differentially expressed genes in Nile tilapia gills following the acute challenge with *Flavobacterium davisii*.

Enrichment analysis of the above DEGs showed that the significantly enriched GO terms included respiratory chain transport (potentially associated with oxidative stress) and regulation of protein synthesis (Figure 3). Several GO terms were similar in terms of the number of genes and the adjusted *p* value between the three timepoints. A good example is those GO terms related to ubiquitin regulation. However, distinct GO terms were also observed, such as chromatin organization and regulation of the protein modification process at 12 h, indicating ongoing changes in gene and protein regulation. 

**Figure 4:**
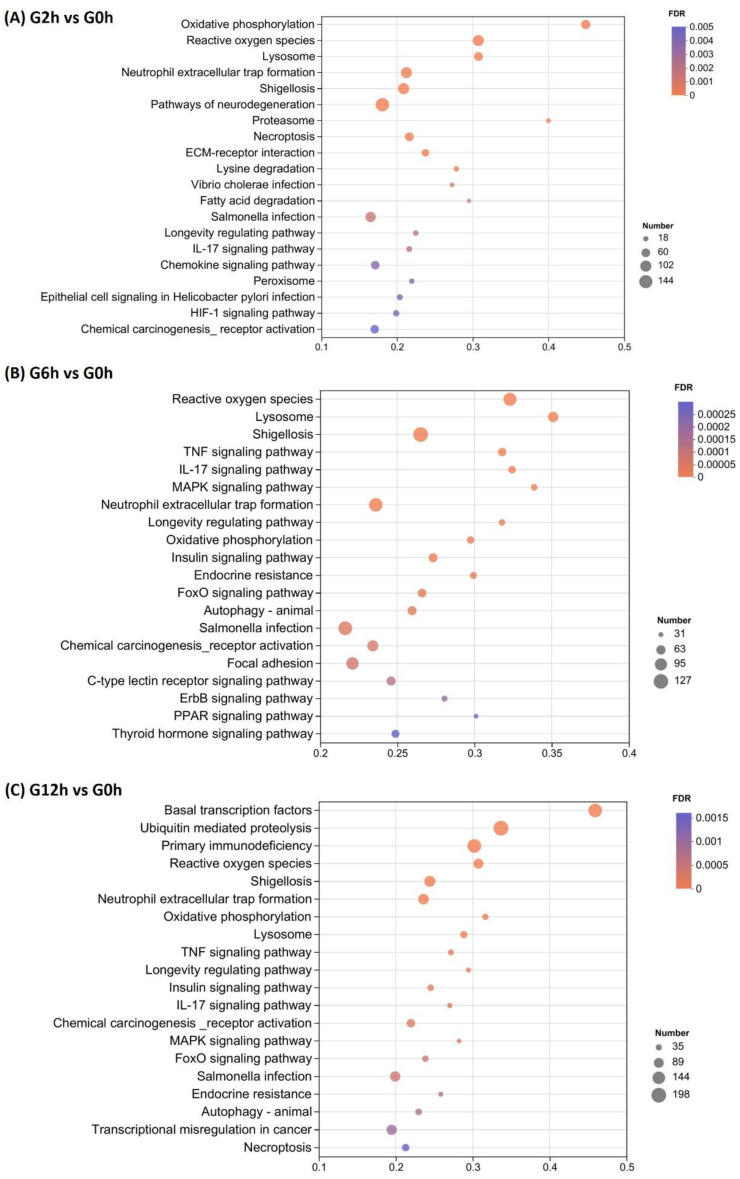
The top 20 significantly enriched Kyoto Encyclopedia of Genes and Genomes (KEGG) pathways in Nile tilapia gills at 2, 6, and 12 h after the acute challenge with *Flavobacterium davisii* compared with the control (0 h). (**A**) 2 h vs. 0 h. (**B**) 6 h vs. 0 h. (**C**) 12 h vs. 0 h.

The top 20 significantly enriched KEGG pathways included those related to oxidative stress, lysosomes, immune suppression, bacterial infection, and tissue necrosis, among other relevant signaling pathways (Figure 4). At 2 h, the top three enriched KEGG pathways included the pathways of neurodegeneration, reactive oxygen species, and neutrophil extracellular trap formation. At 6 h, reactive oxygen species and neutrophil extracellular trap formation were still among the top three enriched KEGG pathways besides shigellosis. Changes in the enriched KEGG pathways were evident at 12 h with the top three including basal transcription factors, ubiquitin-mediated proteolysis, and primary immunodeficiency, suggesting possible host responses to regulate gene expression. 

**Figure 5:**
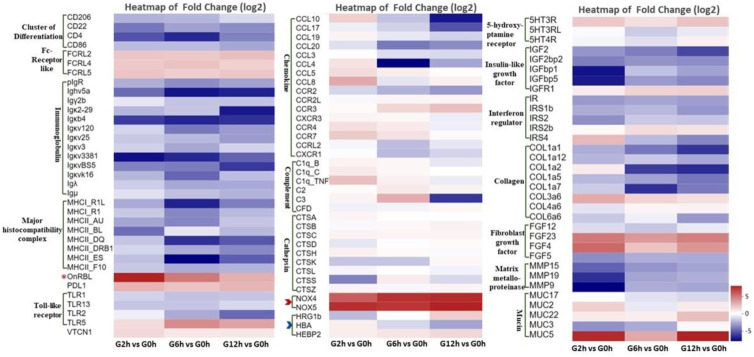
Heatmap of fold change (log2) of differentially expressed genes (DEGs) in Nile tilapia gills at 2, 6, and 12 h after the acute challenge with *Flavobacterium davisii* compared with the control (0 h). The star represents the significantly expressed rhamnose-binding lectin (OnRBL). The red arrow indicates oxidative stress-related genes, while the blue arrow indicates oxygen transport genes. Please refer to the list of gene abbreviations (Table S4) in the Supplementary Materials for the full gene names.

According to functional enrichment, pathway analysis, manual annotation, and literature searches, the key DEGs were classified into several categories, including immune response (major histocompatibility complex (MHC), immunoglobulins (Ig), chemokines, complements, Fc receptor-like genes (FcRLs), Toll-like receptors (TLRs), etc.), oxidative stress, oxygen transport, apoptosis, tissue remodeling (collagen, fibroblast growth factor, and matrix metalloproteinase), and neuroendocrine-related (Figure 5; Table S3). The upregulated DEGs included FcRLs, immunosuppressive programmed death-ligand 1 (PDL1), the nuclear receptor corepressor 1 (NCoR1), calcium-calmodulin-dependent protein kinase II beta (CAMK2B), nicotinamide adenine dinucleotide phosphate (NADPH) oxidase 4 (NOX4), NOX5, rhamnose-binding lectin (RBL), mucin genes (MUC2, MUC3A, MUC5AC, MUC17), serotonin receptors (5HT3R, 5HT3RL, 5HT4R), and insulin-like growth factor-related genes. On the other hand, most immune-related genes were downregulated, including those related to lymphocyte activity such as TLRs, MHC, Ig, chemokines, and complement genes. Putative functional roles and the significance of these gene categories/pathways are covered in depth in the Section 4. 

### 3.4. qPCR Validation

**Figure 6:**
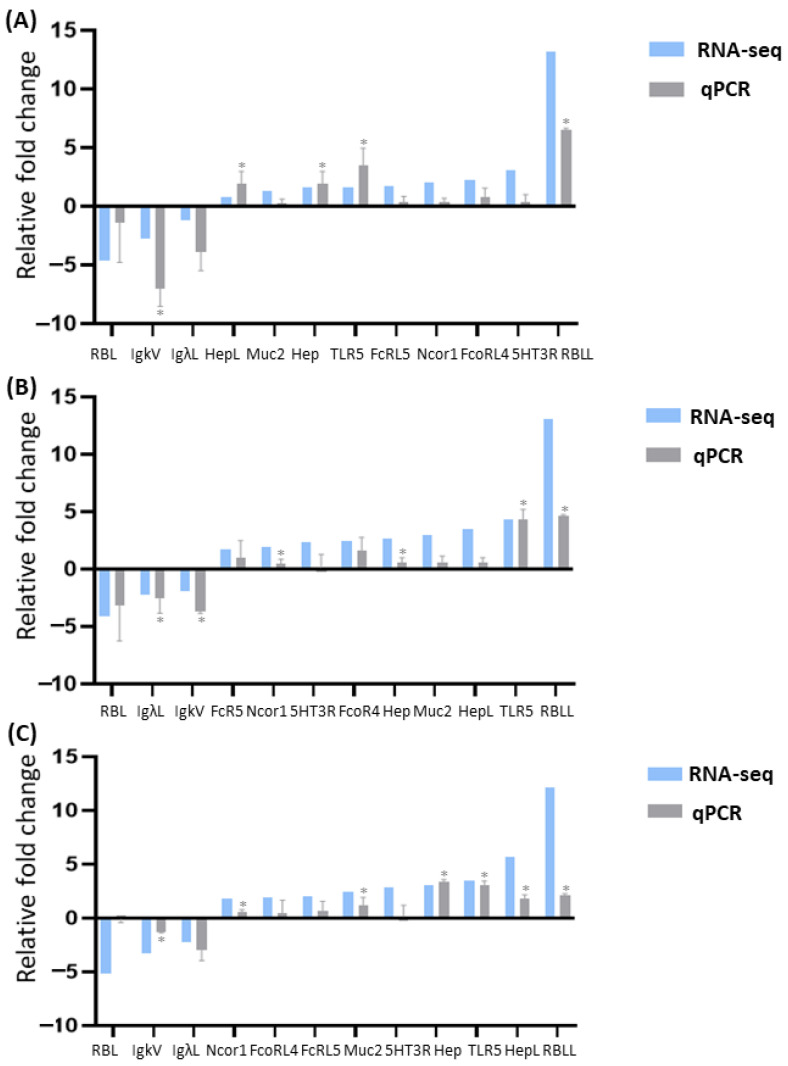
qPCR validation of differentially expressed genes (DEGs) identified by RNA-seq in Nile tilapia gills acutely challenged with *Flavobacterium davisii*. DEGs were validated at three timepoints: 2 h (**A**), 6 h (**B**), and 12 h (**C**) post-infection. Note: The asterisk (*) indicates statistically significant difference in gene expression between the challenged fish and the control group.

Twelve genes based on functional enrichment and pathway results were selected to validate the DEGs identified by RNA-seq using qPCR. Melting-curve analysis revealed a single product for all tested genes. Fold changes from qPCR were compared with the RNA-seq expression analysis results. The qPCR results were significantly correlated with the RNA-seq results at each timepoint (high correlation coefficients 0.93, 0.92, and 0.93, respectively; *p*-value < 0.01; Figure 6). All examined genes had the same trend of differential expression by both methods, indicating the high reliability and accuracy of our RNA-seq-based transcriptome analysis.

## 4. Discussion

This study conducted a transcriptomic analysis of gill tissues in tilapia experiencing an acute infection with a highly virulent *F. davisii* strain, aiming to explore the distinctive changes in the expression of immune-related genes in the gill mucosa. Results from the enrichment analysis of DEGs post-infection revealed a significant enrichment of genes associated with respiratory chain transport and the regulation of protein synthesis, among other relevant GO term gene sets. KEGG pathway analysis indicated that some differentially expressed genes were enriched in signaling pathways related to oxidative stress, lysosomes, immune suppression, bacterial infection, and tissue necrosis. These findings not only provide valuable insights into columnaris disease progression, but also set the stage for subsequent gene categorization and a more in-depth analysis of immune response mechanisms.

Further functional classification indicated that the immune response mechanism was not effectively triggered in the acutely challenged fish. Most immune genes were suppressed, especially those related to lymphocyte activity, including MHC and Ig genes. TLRs, chemokines, complement genes, and lysosomal protease genes with immunostimulatory or immune defense roles were significantly downregulated. Immune evasion-related genes, such as FcRLs, and PDL1 were significantly upregulated [28]. These presented the anti-immune strategies of *F. davisii*, including blocking acquired immunity, stopping or preventing TLR signaling, and inhibiting complement cytokines/chemokines, as indicated in many bacterial pathogens [29,30]. Immune suppression of early host immune responses by the virulent isolate of *Flavobacterium covae* has been reported in catfish [30], but the related pathways and genes are different. These differences may be attributed to bacterial species, host species, fish age, and health status. Immune repression is a common mechanism for pathogens to evade host immune surveillance and clearance [4,5,30].

The present study demonstrated a two-fold upregulation of NCoR1 in Nile tilapia following the acute challenge with *F. davisii* (Table S3). NCoR1 represses the expression of genes, including immune response genes, through interaction with histone deacetylases. This interaction enhances chromatin condensation into a tighter-packed status and blocks access to the transcription machinery [31]. A recent study revealed similar results in channel catfish fry, where NCoR1 was upregulated in the gills following a challenge with a virulent *F. covae* strain [30]. Activation of calcium-calmodulin-dependent protein kinase (CAMKII) antagonizes the effect of NCoR1 [32]. Once activated, CAMKII recruits ubiquitylation machinery for NCoR1 removal and the resumption of gene transcription. Our results revealed a three-fold upregulation of CAMK2B, which may indicate ongoing modulatory actions to counteract immune gene repression by NCoR1. 

The reactive oxygen species (ROS)-producing genes (NOX4 and NOX5) were significantly upregulated, which can cause substantial tissue damage and hypoxia (as evidenced by the significant downregulation of oxygen transport-related genes such as hemoglobin A (HBA) and heme-binding protein 2 (HEBP2)). The bactericidal mechanism induced by the virulent strain’s ROS production causes substantial tissue damage. Our study results also demonstrated significant upregulation of genes related to extracellular matrix degradation and repair. Previous research has indicated a remarkable downregulation of genes associated with the extracellular matrix, cytoskeleton, and tissue repair in mucosal tissues of channel catfish (*Ictalurus punctatus*) following infection with virulent strains [9,33]. This is associated with the ability of bacteria to produce connective tissue-degrading enzymes, chondroitinases, and proteases, which damage fish tissues. In contrast, in resistant fish (those vaccinated with attenuated vaccines or treated with immunostimulants), the expression of these genes upon *F. columnare* infection was significantly higher than in the control group, promoting tissue repair and reducing mortality rates [9,34].

Most importantly, the overexpression of *Oreochromis niloticus* RBL (OnRBL) and mucin genes in the early infection times implied their crucial roles in facilitating bacterial adhesion. During this process, bacterial adhesion to the host is the first step for successful infection. Bacteria utilize the host’s immune responses to enhance adhesion, thereby aiding in successful infection [7,8,9,10]. The expression of RBL in the gill tissue of tilapia is closely related to the infection intensity (mortality rate) during the early stages of infection. Our preliminary research revealed no significant difference in RBL expression at lower infection intensities. However, RBL expression was significantly upregulated at higher infection intensities (peaking at two hours and then gradually decreasing). It has been found that *F. columnare* can “hijack” RBL protein in the gill tissue of channel catfish to achieve early adhesion and invasion, with this gene being specifically highly expressed during the early stages of infection [8]. The bacteria may utilize sugar ligands on their surface to bind with RBL, helping them evade host immune recognition and thereby facilitating infection [2]. One way to confirm the roles of RBL during columnaris disease progression is gene knockout. Researchers successfully knocked out the RBL gene in channel catfish using the CRISPR-Cas9 system, inducing a variety of deletion and frameshift mutations that should block the RBL function [35]. However, the phenotypic consequences of RBL knockout on columnaris disease progression or resistance still need to be investigated. Moreover, pre-treatment of fish with the RBL ligand, rhamnose, through immersion has been shown to significantly reduce the mortality rate due to *F. columnare* infection, demonstrating a significant disease-blocking effect while simultaneously inhibiting the expression of the RBL gene [7]. However, whether rhamnose pre-treatment can block *F. davisii* infection in tilapia requires further investigation. 

The current results demonstrated an upregulation of several mucin-related genes following *F. davisii* infection. Among these genes are MUC2, MUC3A, MUC5AC, and MUC17. Mucins, the main component of mucus, are heavily glycosylated high molecular weight proteins that play a vital role in immune response, inflammation, and bacterial adhesion. *F. columnare* exhibits chemotaxis to mucus and can stimulate the secretion of mucins in fish [29]. A recent study reported the upregulation of eight mucin-related genes in channel catfish four hours after a challenge with *F. columnare* at a concentration of 3 × 10^6^ CFU/ml [36]. Previous research on channel catfish demonstrated a correlation between mucin-related gene expression and susceptibility to columnaris disease, with higher expression levels evident in columnaris-susceptible catfish than in columnaris-resistant fish [29]. 

In the current study, serotonin (5-hydroxytryptamine) receptors (5HT3R, 5HT3RL, and 5HT4R) were upregulated in the challenged fish compared with the control group. Serotonin is one of the most important neurotransmitters in the central nervous system of vertebrates. Serotonin acts centrally and peripherally, controlling several functions, such as cognition, behavior, and emotions, besides acting as a peripheral hormone in various bodily organs [37]. In mammals, serotonin plays essential immunomodulatory roles in immune cells, including macrophages and monocytes [37]. The immunomodulatory roles of serotonin during bacterial infection have also been reported in teleosts [38]. For example, intraperitoneal inoculation of Patagonian blennie (*Eleginops maclovinus*) juveniles with 100 µL of *Francisella noatunensis* culture at concentrations ranging from 1.5 × 10^1^ to 1.5 × 10^10^ bact/µL stimulated higher brain serotonin levels than the control group, implying a possible immunomodulatory role of serotonin in fish during bacterial infection [39]. A recent study investigated the immunomodulatory effects of the serotonin system in Nile tilapia following infection with *Streptococcus agalactiae* [40]. The researchers cloned 22 conserved serotonergic genes and assessed their expression at 0 h, 6 h, 12 h, 24 h, and 48 h after the challenge with *S. agalactiae*. Their results indicated induced expression of different marker genes in various tissues, with On-HTR3A displaying the highest expression in the gills [40]. 

The acute challenge of Nile tilapia with *F. davisii* upregulated the insulin-like growth factor (IGF)-related genes, including insulin-like growth factor 1 receptor, insulin-like growth factor 2 binding protein 2, insulin-like growth factor binding protein 7, insulin-induced gene 1 and 2, and insulin receptors. The most common roles of IGF-1 include regulating fish growth, reproduction, and osmoregulation [41]. However, studies have indicated its potential immunomodulatory function as well. In vivo, the administration of recombinant salmon IGF-1 in rainbow trout (*Oncorhynchus mykiss*) elevated their plasma lysozyme levels in a concentration-dependent manner [42]. In golden pompano (*Trachinotus ovatus*), insulin-like growth factor binding protein 3 (IGFBP3) exhibited antimicrobial activity against *Vibrio harveyi,* as demonstrated by the significantly lower bacterial loads in the liver, spleen, and head kidney of treated fish compared with the control group [43]. These results highlight the important regulatory roles of relevant hormones and neurotransmitters in the immune response mechanisms of Nile tilapia. 

## 5. Conclusions

In conclusion, this research elucidates the expression response characteristics of immune-related genes in the gill mucosa of Nile tilapia induced by *F. davisii*. It reveals that *F. davisii* enhances its own adhesion, leading to immune suppression and tissue damage in the host, thereby achieving successful infection. These findings provide a theoretical basis for understanding *F. davisii* pathogenesis and developing prevention and control strategies. Future research should explore chronic exposure to lower *F. davisii* doses and investigate the long-term transcriptomic changes. Comparing acute and chronic exposure results would help identify more pivotal genes for immune response modulations and resistance to columnaris disease in Nile tilapia.

## Data Availability

All data supporting the reported results are included within the manuscript.

## Supplementary Materials

The following supporting information can be downloaded at: https://www.mdpi.com/article/10.3390/biology13110894/s1, Table S1: Primer sequences used for qPCR validation; Table S2: Statistical table of RNA sequencing data; Table S3: Significantly differentially expressed genes in the gills of Nile tilapia post *Flavobacterium davisii* challenge at 2, 6 and 12 h compared to the control (0 h) respectively title; Table S4: List of gene abbreviations.
