# Supplementary material for: Immune Suppression and Rapid Invasion of Nile Tilapia Gills Following an Acute Challenge by Flavobacterium davisii
Source: Biology (Basel). 2024 Nov 2;13(11):894. doi: 10.3390/biology13110894 (PMC11592246; doi:10.3390/biology13110894)
Supplement: Supplementary file 1 [file biology-13-00894-s001.zip › biology-3256208-S1,S2,S4.pdf]

Table S1. Primer sequences used for qPCR validation.

Gene name	Primers (5' to 3')
β-actin-F	AACAACCACACACCACACATTTC
β-actin-R	TGTCTCCTTCATCGTTCCAGTTT
5HTR3-F	CCGTCCATCACAGTCCAGTC
5HTR3-R	CAGCGAGTCAATCCAGTCCAA
FcR4L-F	CCACACCTCCACCTTCATCC
FcR4L-R	AGTCTCACCTTCCAGCCATCT
FcR5L-F	ACTTGTCGGTGATGGTTGATGA
FcR5L-R	TGCCTTGAGCGGTGTTCTG
Hepcidin-F	GCCGTCGTGCTCACATTCA
Hepcidin-R	GCTCCTGCTCCTGCTAATGAA
Hepcidin-like-F	TTGCCAGGACGCTGAGATG
Hepcidin-like-R	CCATTGATGACTCCGCTGACA
Ig λ L-F	GCTGCTTGTTGCTGCTGTC
Ig λ L-R	GGTTCTCCGCCGTTCTTCTT
IgκV-F	CAGGCTGAAGATGCTGGAGAT
IgκV-R	TCTGCCAGACTGAAGGAGGT
Mucin2-F	ACAGCATCTCCGACAACAACA
Mucin2-R	AGCACGCAGCCTTCATTCT
Ncor1-F	CTCCTCCACACTCCTCAGACT
Ncor1-R	TCCATTCGCTCCACCTTCTTC
RBL-F	CCGTACTTCTGATCCGTG CTA
RBL-R	CTGAGAGGCTGGACGATTGAA
RBL-like-F	GCAGATGTGATGGCAAGAAGG
RBL-like-R	GCTGAGAGGCTGGAAGATTCA
TLR5-F	TGACTGCAATCTGGGGAACT
TLR5-R	ACTTTAGACCTCGGACTGCC

Table S2. Statistical table of sequencing data.

Sample	Raw reads	Clean reads	Clean bases	Error rate (%)	Q20 (%)	Q30 (%)	GC content (%)
G0h_rep1	66677810	66279680	9820329558	0.0251	98.05	94.1	48.2
G0h_rep2	50360024	49979940	7370754317	0.0247	98.15	94.46	48.59
G2h_rep1	72620748	71736166	8383840486	0.0256	98.02	94.15	51.71
G2h_rep2	80907958	79745644	9567528829	0.0256	97.97	94.07	50.99
G6h_rep1	55658736	55235036	7393216327	0.0254	97.99	94.04	50.86
G6h_rep2	76155880	75100418	8692687713	0.0256	98.03	94.22	52.16
G12h_rep1	68178764	67246988	7606564776	0.0262	97.85	93.8	52.47
G12h_rep2	81006170	79523290	8476080040	0.026	97.95	94.07	52.43

Table S4. List of gene abbreviations.

Gene abbreviation	Gene description
5HT3R	5-hydroxytryptamine receptor 3A
5HT3RL	5-hydroxytryptamine receptor 3A-like
5HT4R	5-hydroxytryptamine receptor 4
AP1	activating protein-1
C1q_B	complement C1q subcomponent subunit B
C1q_C	complement C1q subcomponent subunit C
C1q_TNF	complement C1q tumor necrosis factor-related protein 1
C2	complement C2
C3	complement C3
CFD	complement factor D
CCL10	C-X-C motif chemokine 10
CCL17	C-C motif chemokine 17
CCL19	C-C motif chemokine 20
CCL20	C-C motif chemokine 20
CCL3	C-C motif chemokine 3
CCL4	C-C motif chemokine 4
CCL5	C-C motif chemokine 5
CCL8	C-C motif chemokine 8
CCR2	C-C chemokine receptor type 2-like, transcript variant X1
CCR2	C-X-C chemokine receptor type 2
CCR2L	C-X-C chemokine receptor type 2-like, transcript variant X2
CCR3	C-C chemokine receptor type 3, transcript variant X1
CXCR3	C-X-C chemokine receptor type 3-2
CCR4	C-X-C chemokine receptor type 4, transcript variant X1
CCR7	C-C motif chemokine receptor 7
CCRL2	chemokine-like receptor 1
CXCR1	chemokine XC receptor 1
CD206	CD226 antigen-like
CD22	B-cell receptor CD22-like
Ig λ L	Immunoglobulin lambda-1 light chain
lgκV	Immunoglobulin kappa chain V region
NCOR1	nuclear receptor corepressor 1
CD4	programmed cell death 1 ligand 1-like, transcript variant X1
CD86	CD86 molecule, transcript variant X1
CEACAM5	carcinoembryonic antigen-related cell adhesion molecule 5
COL1a1	collagen alpha-1(I) chain-like
COL1a12	collagen alpha-1(XII) chain, transcript variant X1
COL1a2	collagen alpha-1(II) chain, transcript variant X1
COL1a5	collagen alpha-1(V) chain, transcript variant X2
COL1a7	collagen type VII alpha 1 chain, transcript variant X2
COL3a6	collagen alpha-3(VI) chain-like
COL4a6	collagen alpha-4(VI) chain
COL6a6	collagen alpha-6(VI) chain
CTSA	lysosomal protective protein
CTSB	cathepsin B
CTSC	cathepsin C
CTSD	cathepsin D
CTSH	cathepsin H
CTSK	cathepsin K-like

CTSL	cathepsin L1, transcript variant X1
CTSS	cathepsin S
CTSZ	cathepsin Z
DDIT4	DNA damage inducible transcript 4
DDIT4L	DNA damage-inducible transcript 4 protein
FcRL2	Fc receptor-like B, transcript variant X1
FcRL4	Fc receptor-like protein 4, transcript variant X1
FcRL5	Fc receptor-like protein 5
FOS2	FOS like 2, AP-1 transcription factor subunit
FOSb	FosB proto-oncogene, AP-1 transcription factor subunit, transcript variant X2
GAL3	galectin-3-binding protein A
GRN	granulins
GZMa	granzyme A-like
GZMb	granzyme B-like
GZMk	granzyme K-like
HBA	hemoglobin subunit beta-A-like, transcript variant X1
HEBP2	heme-binding protein 2
HIC1	HIC ZBTB transcriptional repressor 1, transcript variant X1
HIC2	HIC ZBTB transcriptional repressor 2, transcript variant X2
HO	heme oxygenase
HRG1b	heme transporter hrg1-B
pIgR	polymeric immunoglobulin receptor
Ighv5a	Ig heavy chain V region 5A-like
Ig γ 2b	Ig gamma-2B chain C region-like
Ig κ 2-29	immunoglobulin kappa variable 2-29
Ig κ b4	Ig kappa-b4 chain C region-like
Ig κ v120	Ig kappa chain V region 120-like
Ig κ v25	Ig kappa chain V region K-25-like
Ig κ v3	Ig kappa chain V-III region MOPC 63-like isoform X1
Ig κ v3381	Ig kappa chain V region 3381-like
IgκvBS5	Ig kappa chain V region BS-5-like
Ig λ	immunoglobulin lambda-1 light chain
Ig μ	Ig mu chain C region membrane-bound form-like isoform X2
IL1R1	interleukin-1 receptor-like 1, transcript variant X1
IL1R2	interleukin-1 receptor type 2, transcript variant X1
IL1R2L	interleukin-1 receptor type 2-like
IRAK1	interleukin 1 receptor associated kinase 1
MHCI_R1L	major histocompatibility complex class I-related gene protein-like
MHCI_R1	major histocompatibility complex class I-related gene protein
MHCII_AU	H-2 class II histocompatibility antigen, A-U alpha chain
MHCII_BL	class II histocompatibility antigen, B-L beta chain
MHCII_DQ	HLA class II histocompatibility antigen, DQ beta 1 chain-like, transcript variant X1
MHCII_DRB1	HLA class II histocompatibility antigen, DRB1-8 beta chain-like
MHCII_ES	H-2 class II histocompatibility antigen, E-S beta chain
MHCII_F10	class I histocompatibility antigen, F10 alpha chain, transcript variant X1
NFκB_id	NFKB inhibitor delta
NOX4	NADPH oxidase 4, transcript variant X1
NOX5	NADPH oxidase 5, transcript variant X1
OnRBL	L-rhamnose-binding lectin CSL2-like
PDL1	programmed cell death 1 ligand 1

TLR1	toll-like receptor 1
TLR13	toll-like receptor 13
TLR2	toll-like receptor 2
TLR2	toll-like receptor 2
TLR5	toll-like receptor 5
VTCN1	V-set domain containing T-cell activation inhibitor 1
